# Prognostic impact of glucagon-like peptide-1 receptor (GLP1R) expression on cancer survival and its implications for GLP-1R agonist therapy: an integrative analysis across multiple tumor types

**DOI:** 10.1007/s11357-024-01494-5

**Published:** 2025-01-08

**Authors:** Zoltan Ungvari, Áron Bartha, Anna Ungvari, Monika Fekete, Giampaolo Bianchini, Balázs Győrffy

**Affiliations:** 1 Vascular Cognitive Impairment, Neurodegeneration and Healthy Brain Aging Program, Department of Neurosurgery, University of Oklahoma Health Sciences Center, Oklahoma City, OK USA; 2 Stephenson Cancer Center, University of Oklahoma, Oklahoma City, OK USA; 3 Oklahoma Center for Geroscience and Healthy Brain Aging, University of Oklahoma Health Sciences Center, Oklahoma City, OK USA; 4 Department of Health Promotion Sciences, College of Public Health, University of Oklahoma Health Sciences Center, Oklahoma City, OK USA; 5 International Training Program in Geroscience, Doctoral College/Institute of Preventive Medicine and Public Health, Semmelweis University, Budapest, Hungary; 6 Dept. of Bioinformatics, Semmelweis University, 1094 Budapest, Hungary; 7 Institute of Preventive Medicine and Public Health, Semmelweis University, Budapest, Hungary; 8 Department of Medical Oncology, IRCCS Ospedale San Raffaele, Milan, Italy; 9 Dept. of Biophysics, Medical School, University of Pecs, H-7624 Pecs, Hungary; 10 Cancer Biomarker Research Group, Institute of Molecular Life Sciences, HUN-REN Research Centre for Natural Sciences, 1117 Budapest, Hungary

**Keywords:** GLP-1 receptor agonists, Cancer prognosis, Gene expression, Survival analysis, Tumor biomarkers, Type 2 diabetes treatment, KM plotter

## Abstract

Glucagon-like peptide-1 receptor (GLP-1R) agonists, such as exenatide (Byetta, Bydureon), liraglutide (Victoza, Saxenda), albiglutide (Tanzeum), dulaglutide (Trulicity), lixisenatide (Lyxumia, Adlyxin), semaglutide (Ozempic, Rybelsus, Wegovy), and tirzepatide (Mounjaro, Zepbound), are widely used for the treatment of type 2 diabetes mellitus (T2DM) and obesity. While these agents are well known for their metabolic benefits, there is growing interest in their potential effects on cancer biology. However, the role of GLP-1R agonists in cancer remains complex and not fully understood, particularly across different tumor types. This study aimed to evaluate the prognostic significance of GLP1R expression on overall survival across various cancer types. Using a comprehensive analysis of gene expression data and survival outcomes a large cohorts of different tumor types, we employed Cox proportional hazards survival analyses, coupled with false discovery rate determinations, to explore correlations between GLP1R expression and survival. The integrated database included thousands of cancer specimens with available overall survival time and event data from numerous independent cohorts, providing a robust platform for survival analysis. Our findings reveal that increased GLP1R expression is associated with improved overall survival in cancers such as bladder cancer, breast cancer, esophageal adenocarcinoma, renal clear cell carcinoma, and thyroid carcinoma. Conversely, higher GLP1R expression is linked to poorer survival outcomes in cervical squamous cell carcinoma, lung squamous cell carcinoma, stomach adenocarcinoma, and uterine corpus endometrial carcinoma. Additionally, GLP1R expression showed no significant impact on overall survival in cancers such as esophageal squamous cell carcinoma, colon cancer, head-neck squamous cell carcinoma, renal papillary cell carcinoma, hepatocellular carcinoma, lung adenocarcinoma, ovarian cancer, and pancreatic cancer. In conclusion, GLP1R expression levels serve as an important biomarker with potential prognostic significance across multiple cancers, demonstrating both protective and adverse associations depending on the tumor type. These findings highlight the complex role of GLP-1R agonists in cancer risk and survival, suggesting that the therapeutic use of these agents should be carefully tailored to the individual patient’s cancer risk profile.

## Introduction

Glucagon-like peptide-1 (GLP-1) receptor agonists have emerged as a pivotal class of medications primarily used in the treatment of type 2 diabetes mellitus (T2DM) and obesity [1, 2]. Approved GLP-1 agonists include exenatide (Byetta, Bydureon), liraglutide (Victoza for diabetes, Saxenda for obesity), albiglutide (Tanzeum), dulaglutide (Trulicity), lixisenatide (Lyxumia in Europe, Adlyxin in the U.S.) and semaglutide (Ozempic, Rybelsus for diabetes, Wegovy for obesity). Additionally, tirzepatide, a dual GLP-1 and GIP agonist (Mounjaro for diabetes, Zepbound for obesity) is another recent addition to this class of drugs. These agents function by mimicking the actions of endogenous GLP-1, a hormone that is secreted by the gut in response to food intake [1, 2]. The activation of the GLP-1 receptor (GLP1R) triggers several physiological responses, including enhanced insulin secretion, inhibition of glucagon release, and delayed gastric emptying, all of which contribute to improved glycemic control and reduced body weight [1, 2]. Due to their efficacy, GLP1R agonists are widely prescribed, with millions of patients benefiting from their metabolic effects.

Beyond their well-documented metabolic benefits, there is a growing interest in the potential effects of GLP1R agonists on cancer biology [3–9]. The widespread use of these drugs, combined with the fact that cancer is a leading cause of morbidity and mortality globally, necessitates a deeper understanding of how GLP-1 signaling might influence cancer risk and survival. Emerging data suggest that GLP-1 and its downstream mediators, including fibroblast growth factor-21 (FGF-21), could have significant roles in cancer development and progression [3–5, 8, 10–40]. However, the relationship between GLP1R activation and cancer is complex, with some studies suggesting protective effects while others indicate potential risks [8].

Recent data highlight this complexity. A large-scale epidemiological studycompared the incidence of obesity-related cancers in patients treated with GLP1R agonists versus those treated with insulin or metformin [3]. It found a significant reduction in the risk of several cancers among GLP1R agonist users compared to insulin users, but no clear benefit compared to metformin, and even a potential increased risk of kidney cancer [3]. This raises critical questions about whether these observed effects are directly due to GLP-1 receptor activation or are secondary consequences of weight loss and other metabolic changes induced by these drugs. The limitations of this study, including its retrospective design and the difficulty in separating direct drug effects from the consequences of weight loss, underscore the need for further investigation.

This study was designed to evaluate the prognostic significance of GLP1R expression across various cancer types, with the hypothesis that GLP1R expression might serve as an indicator of certain cancers’ sensitivity to glucagon/GLP-1 signaling. We hypothesized that patients with tumors exhibiting high GLP1R expression could experience distinct survival outcomes, potentially reflecting their tumors’ responsiveness to GLP1R activation. To test this hypothesis, we conducted a comprehensive analysis of survival data across multiple cancer types, using integrated cancer patient cohorts from the Kaplan–Meier Plotter platform [41–43]. This approach allowed us to examine the relationship between GLP1R expression levels and patient outcomes, and to contextualize these findings within the broader landscape of clinical data on GLP1R agonist use [41, 42].

## Methods

### Differential gene expression across multiple tissue types

We employed the database from the previously established TNMplot project to compare gene expression levels between tumor and normal samples across multiple datasets [44]. In this platform we have integrated data from The Cancer Genome Atlas (TCGA) and The Genotype-Tissue Expression (GTEx) repositories. RNAseq based gene expression data was processed as previously described [44] and tumor tissues were compared to non-cancerous tissues from the same organ. Notably, normal whole blood from GTEx was used as the normal tissues for acute myeloid leukemia samples. Each tumor type was processed independently, and the differential gene expression was determined by computing a Mann–Whitney *U*-test. Statistical significance was set at *p* < 0.05. We employed a boxplot for the visualization of the data.

### Survival analysis across different tumor types

Data was collected as previously described for breast [45], colon [46], lung [41], ovarian [47], gastric [48], and pancreatic [49] and other [50] cancers (Table 1).

**Table 1:** Clinical characteristics of the included cancer patients. Note that with the exception of relapse-free survival data, not all patients had all data available

Feature	Bladder carcinoma	Breast cancer	Cervical squamous cell carcinoma	Colorectal cancer	Esophageal Adenocarcinoma	Head-neck squamous cell carcinoma	Kidney renal clear cell carcinoma	Kidney renal papillary cell carcinoma	Liver hepatocellular carcinoma	Lung adenocarcinoma	Lung squamous cell carcinoma	Ovarian cancer	Stomach adenocarcinoma	Thyroid carcinoma	Uterine corpus endometrial carcinoma	Pancreas carcinoma
Number of patients	405	1090	304	454	80	500	530	288	371	513	501	374	375	502	543	177
Sex																
Female	107	1079	304	214	23	133	186	76	121	276	130	374	137	367	543	80
Male	301	12	-	240	141	368	344	213	250	239	371	-	243	135	-	97
Stage																
Definition	AJCC	AJCC	FIGO	AJCC	AJCC	AJCC	AJCC	AJCC	AJCC	AJCC	AJCC	FIGO	AJCC	AJCC	FIGO	AJCC
1	2	181	162	75	17	25	265	172	171	276	244	1	55	281	339	21
2	130	619	69	176	70	71	57	21	86	121	162	21	112	52	51	146
3	140	248	45	128	50	78	123	52	85	84	84	292	151	112	124	3
4	134	20	21	64	8	259	82	15	5	26	7	57	39	55	29	4
Pathological T																
1	3	279	140	11	29	45	271	193	181	169	114	NA	20	143	NA	7
2	119	631	71	77	38	133	69	32	94	277	293	NA	84	164	NA	24
3	194	138	20	309	77	96	179	60	80	47	71	NA	168	170	NA	141
4	58	40	10	56	4	171	11	2	13	19	23	NA	100	23	NA	3
Pathological N																
0	237	514	133	267	67	172	239	50	252	332	319	NA	113	229	NA	49
1	46	361	60	105	64	65	16	24	4	95	131	NA	99	223	NA	123
2	75	120	NA	82	10	164	NA	4	NA	74	40	NA	76	NA	NA	NA
3	8	76	NA	NA	6	7	NA	NA	NA	2	5	NA	74	NA	NA	NA
Pathological M																
0	196	908	116	333	121	187	420	95	266	346	411	NA	334	280	NA	79
1	11	22	10	64	8	1	78	9	4	25	7	NA	26	9	NA	4

We employed Cox proportional hazards regression analysis to assess the correlation between GLP1R and GCG expression and overall survival. As the gene arrays can have multiple probe sets for a selected gene, the probe set 208400_at was selected for GLP1R and 206422_at for GCG in the survival analysis. These probe sets were previously assigned the most representative for these genes [51].

To avoid missing a correlation due to different cutoff values (e.g., because of using a median gene expression as a cutoff), each analysis was performed by using the best available cutoff. In this, each available cutoff value between the lower and upper quartiles of expression were evaluated, and the best performing cutoff was used in the final analysis. False discovery rate was computed to correct for multiple hypothesis testing. In addition to hazard rate (HR), logrank *p* and 95% confidence intervals were also computed. Finally, Kaplan–Meier plots were drawn to visualize the survival differences between those patients who had high expression and those who had low expression of the selected gene.

## Results

### Differential gene expression across different tumor types

**Fig. 1:**
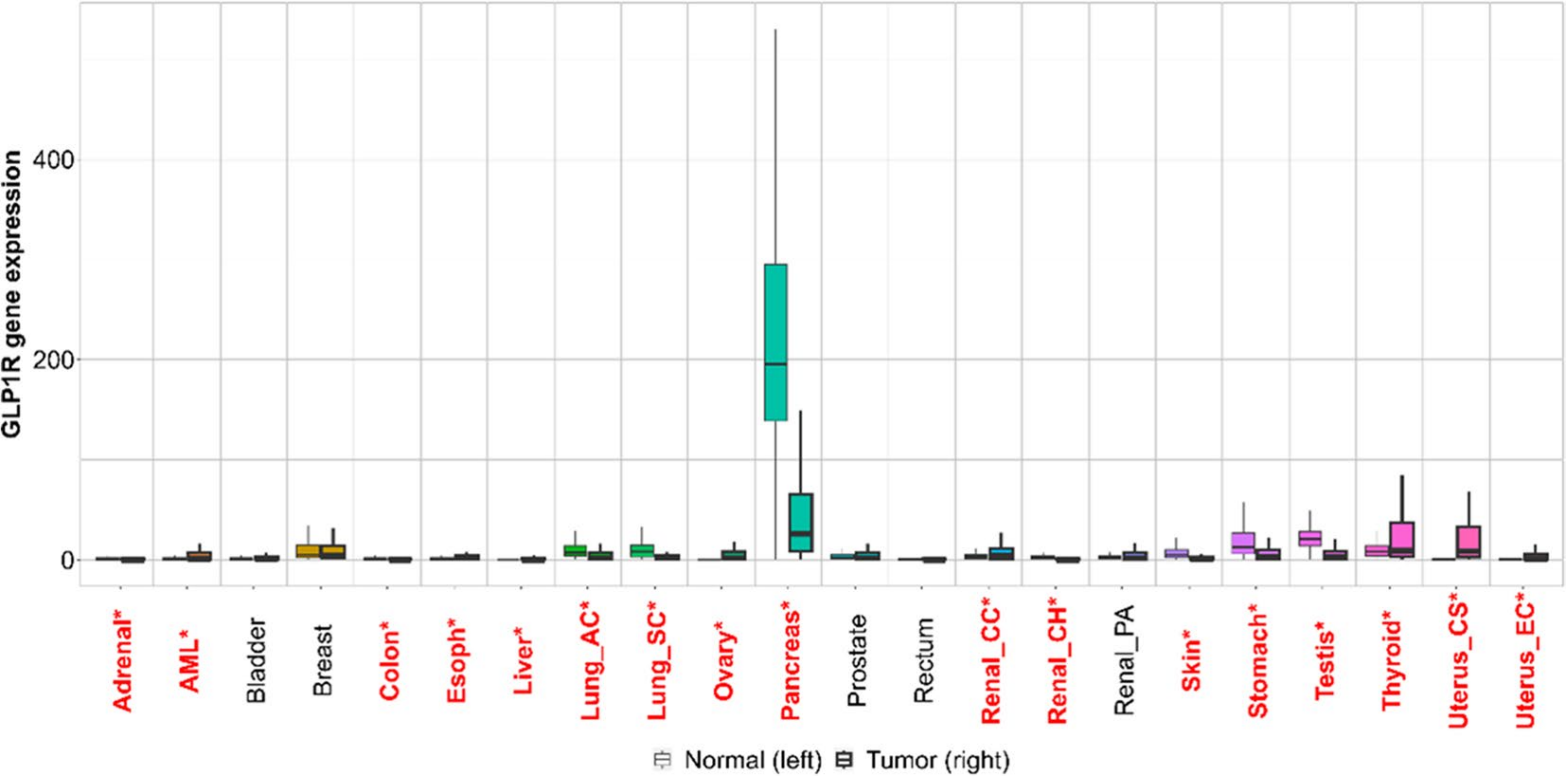
GLP1R gene expression across normal and tumor tissues in various cancer types. The box plot illustrates the mRNA expression levels of GLP1R in a range of normal (left) and tumor (right) tissues across multiple cancer types. The tissues analyzed include the adrenal gland, acute myeloid leukemia (AML; control: while blood from the GTEx), bladder, breast, colon, esophagus, liver, lung (adenocarcinoma and squamous cell carcinoma), ovary, pancreas, prostate, rectum, renal (clear cell carcinoma, chromophobe, papillary adenoma), skin, stomach, testis, thyroid, uterus (cervical squamous cell carcinoma, endocervical adenocarcinoma, endometrial carcinoma), and others. Tissues labeled in red represent statistically significant differences between normal and tumor expressions, with higher or lower GLP1R expression observed in tumor tissues. The black boxes indicate median values, while the whiskers represent the range of expression levels

First, we have analyzed the gene expression levels of GLP1R and GCG across various normal and tumor tissues. GLP1R expression was significantly elevated in pancreatic tumors compared to normal pancreatic tissues, with the median expression reaching around 200 in tumors while remaining close to zero in normal tissues. Additionally, ovarian tissues also exhibited an increase in GLP1R expression in tumor samples compared to normal tissues, although the difference was less pronounced than in the pancreas. In other tissues, including adrenal, AML, colon, liver, lung, and renal tissues, GLP1R expression remained relatively low in both normal and tumor samples, suggesting a tissue-specific overexpression of GLP1R in certain cancers (see Fig. 1).

Similarly, GCG expression was markedly higher in pancreatic tumor tissues, with the median value reaching approximately 35,000, compared to nearly zero in normal pancreatic tissues. This finding aligns with the known role of GCG in pancreatic function and its dysregulation in pancreatic cancer. Prostate and ovarian tumors also exhibited a moderate increase in GCG expression compared to their normal counterparts, but the expression levels were significantly lower than those observed in the pancreas. In most other tissues, GCG expression remained low and consistent between normal and tumor samples, further underscoring the specificity of GCG overexpression in pancreatic cancer. These results highlight the potential of GLP1R and GCG as biomarkers for pancreatic cancer, given their marked overexpression in tumor tissues compared to normal tissues.

### GLP1R expression and survival in different tumor types

**Fig. 2:**
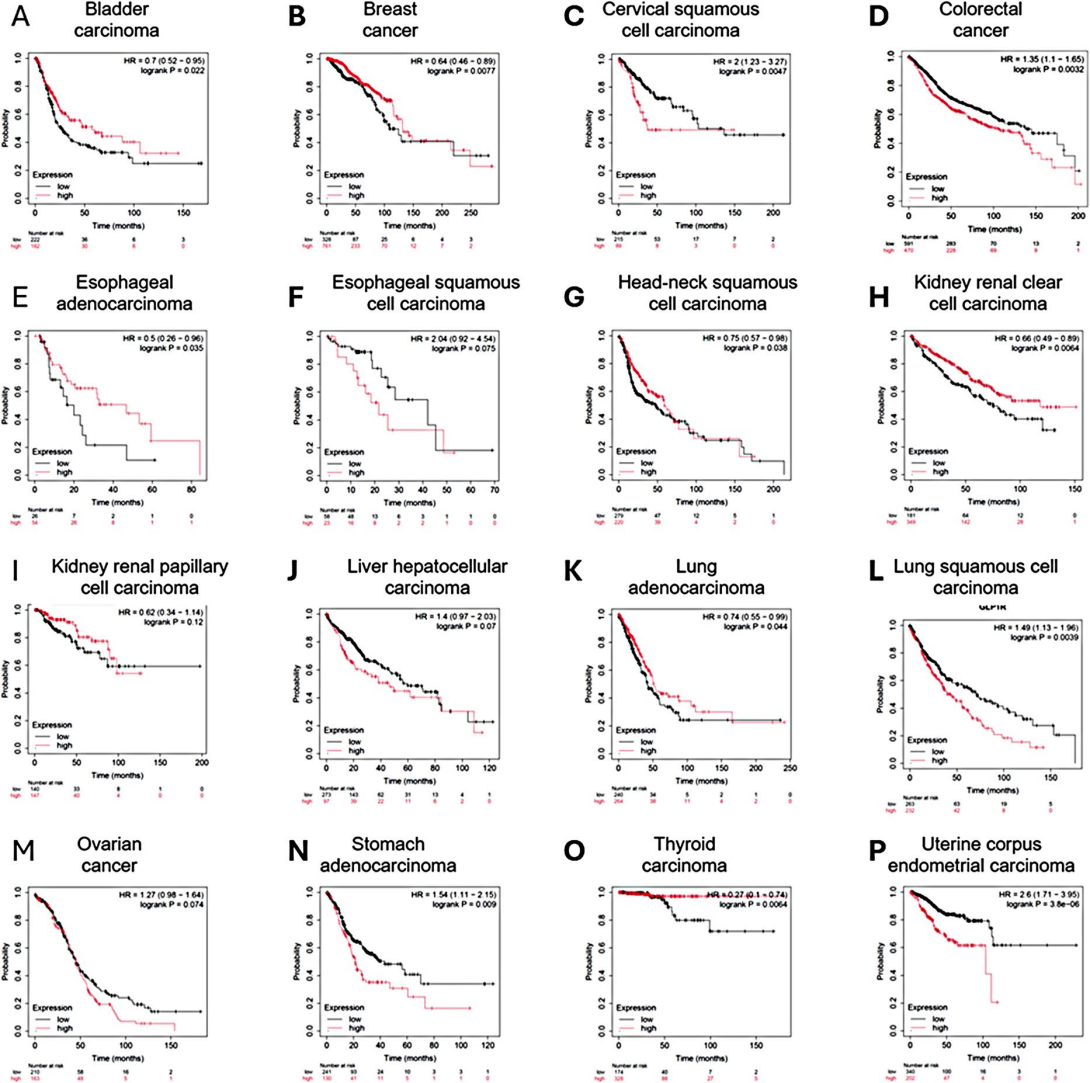
Correlation between GLP1R expression and overall survival across various tumor types. This figure presents Kaplan–Meier survival curves illustrating the correlation between GLP1R expression levels and overall survival in different tumor types. Each panel represents a distinct cancer type, with high (red) and low (black) GLP1R expression groups compared

We analyzed the correlation between overall survival and GLP1R gene expression changes across various cancers. Bladder carcinoma, esophageal squamous cell carcinoma, and lung adenocarcinoma showed no significant difference in survival based on GLP1R expression. In breast cancer, high GLP1R expression was associated with better overall survival (HR = 0.76, logrank *p* = 0.0019), as was the case in esophageal adenocarcinoma (HR = 0.35, logrank *p* = 0.057), renal clear cell carcinoma (HR = 0.73, logrank *p* = 0.0086), kidney renal papillary cell carcinoma (HR = 0.62, logrank *p* = 0.099), and thyroid carcinoma (HR = 0.42, logrank *p* = 0.021). Conversely, higher GLP1R expression correlated with worse survival in cervical squamous cell carcinoma (HR = 2.17, logrank *p* = 0.0074), head-neck squamous cell carcinoma (HR = 1.57, logrank *p* = 0.0063), lung squamous cell carcinoma (HR = 1.47, logrank *p* = 0.0098), stomach adenocarcinoma (HR = 1.31, logrank *p* = 0.026), colon carcinoma (HR = 1.35, logrank *p* = 0.0032), and uterine corpus endometrial carcinoma (HR = 2.36, ogrank *p* < 0.0001). In liver hepatocellular carcinoma, there was a slight improvement in survival with high GLP1R expression (HR = 1.40, ogrank *p* = 0.097). In ovarian cancer, high GLP1R expression was associated with significantly better survival (HR = 1.27, ogrank *p* = 0.14). These findings indicate that GLP1R expression has varying impacts on survival depending on the tumor type, highlighting the complex role of GLP1R signaling in cancer prognosis (see Fig. 2).

### GCG expression and survival in different tumor types

**Fig. 3:**
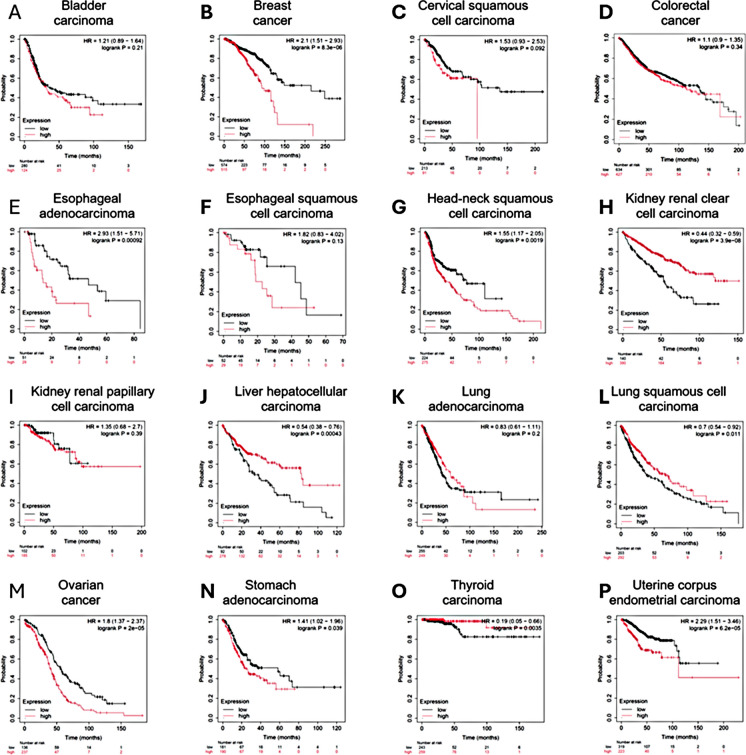
Correlation between GCG expression and overall survival across various tumor types. This figure presents Kaplan–Meier survival curves showing the correlation between GCG expression levels and overall survival in different tumor types. Each panel represents a specific cancer type, comparing high (red) and low (black) GCG expression groups

In Fig. 3, we present Kaplan–Meier survival curves illustrating the correlation between GCG expression levels and overall survival across various tumor types, comparing groups with high (red) and low (black) GCG expression. No significant difference in survival was observed for bladder carcinoma, esophageal squamous cell carcinoma, liver hepatocellular carcinoma, colon cancer, or lung adenocarcinoma based on GCG expression. In contrast, high GCG expression was significantly associated with poorer survival in breast cancer (HR = 2.15, logrank *p* < 0.001), cervical squamous cell carcinoma (HR = 1.83, logrank *p* = 0.0008), esophageal adenocarcinoma (HR = 2.91, logrank *p* = 0.0038), head-neck squamous cell carcinoma (HR = 1.65, logrank *p* = 0.0015), lung squamous cell carcinoma (HR = 1.74, logrank *p* = 0.0011), ovarian cancer (HR = 1.81, logrank *p* = 0.0073), stomach adenocarcinoma (HR = 1.81, logrank *p* = 0.009), and uterine corpus endometrial carcinoma (HR = 2.35, logrank *p* < 0.0001). Conversely, higher GCG expression was correlated with better survival in renal clear cell carcinoma (HR = 0.43, logrank p < 0.001) and thyroid carcinoma (HR = 0.59, logrank *p* = 0.045), with a similar trend observed in renal papillary cell carcinoma (HR = 0.43, logrank *p* = 0.17).

### Gene expression and prognostic implications of GLP1R and GCG in pancreatic cancer

**Fig. 4:**
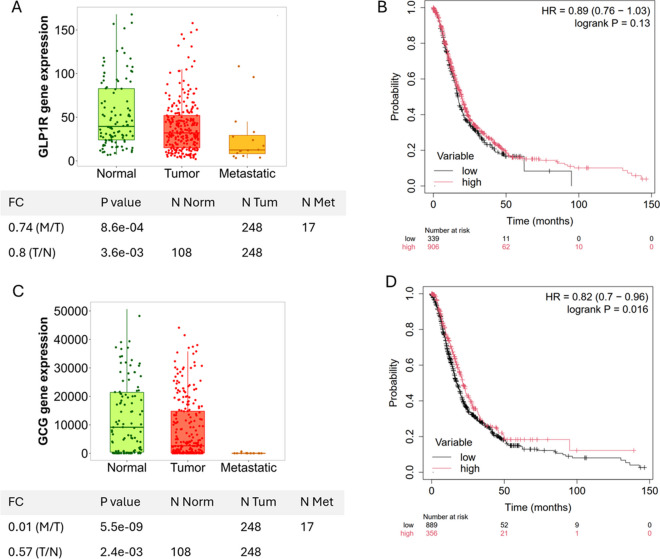
GLP1R and GCG gene expression and their impact on overall survival in pancreatic cancer. This figure presents the gene expression levels of GLP1R and GCG in normal, tumor, and metastatic tissues of pancreatic cancer, along with their correlation with overall survival in patients. Panel A shows that GLP1R expression is significantly lower in pancreatic tumor tissues compared to normal tissues (*p* = 3.64e-03) and further reduced in metastatic pancreatic tissues (*p* = 8.6e-04). Panel B displays the Kaplan–Meier survival curve, indicating that high GLP1R expression is associated with a non-significant trend toward improved survival (HR = 0.89, logrank *p* = 0.13). Panel C illustrates that GCG expression is significantly lower in pancreatic tumor tissues compared to normal tissues (*p* = 2.47e-03), with an even more pronounced reduction in metastatic pancreatic tissues (*p* = 5.57e-09). Panel D shows the Kaplan–Meier survival curve, where high GCG expression is significantly associated with improved overall survival in pancreatic cancer patients (HR = 0.82, logrank *p* = 0.016). These findings suggest that both GLP1R and GCG expressions are reduced in pancreatic tumor and metastatic tissues, with higher expression levels, particularly of GCG, potentially associated with better survival outcomes in pancreatic cancer patients

Figure 4 provides a detailed analysis of the expression levels of GLP1R and GCG genes in normal, tumor, and metastatic pancreatic tissues and their respective correlations with overall survival in pancreatic cancer patients. The results demonstrate a significant reduction in GLP1R expression in tumor tissues compared to normal pancreatic tissues (*p* = 3.64e-03), with even further downregulation observed in metastatic tissues (*p* = 8.6e-04) (Fig. 4, Panel A). Kaplan–Meier survival analysis (Fig. 4, Panel B) indicates a trend toward improved overall survival in patients with higher GLP1R expression (hazard ratio [HR] = 0.89), though this trend did not reach statistical significance (logrank *p* = 0.13). These findings suggest a potential, albeit subtle, influence of GLP1R expression on patient outcomes. Similar to GLP1R, GCG expression was significantly decreased in tumor tissues compared to normal tissues (*p* = 2.47e-03), with a more pronounced reduction in metastatic tissues (*p* = 5.57e-09) (Fig. 4, Panel C). Importantly, Kaplan–Meier survival analysis (Fig. 4, Panel D) revealed a significant association between higher GCG expression and improved overall survival (HR = 0.82, logrank *p* = 0.016), emphasizing its potential role as a prognostic marker in pancreatic cancer.

## Discussion

This study presents a comprehensive analysis of the relationship between GLP1R and GCG expression and overall survival across various cancer types. Our findings demonstrate that increased GLP1R expression is associated with improved overall survival in certain cancers, such as bladder cancer, breast cancer, esophageal adenocarcinoma, renal clear cell carcinoma, and thyroid carcinoma. Conversely, higher GLP1R expression correlates with poorer survival outcomes in cancers such as cervical squamous cell carcinoma, lung squamous cell carcinoma, stomach adenocarcinoma, and uterine corpus endometrial carcinoma. Notably, GLP1R expression appears to have no significant impact on overall survival in cancers such as esophageal squamous cell carcinoma, head-neck squamous cell carcinoma, renal papillary cell carcinoma, liver hepatocellular carcinoma, lung adenocarcinoma, ovarian cancer, and pancreatic cancer. Similarly, increased GCG expression is linked to improved survival in renal clear cell carcinoma, liver hepatocellular carcinoma, lung squamous cell carcinoma, and thyroid carcinoma. However, higher GCG expression is associated with decreased survival in breast cancer, cervical squamous cell carcinoma, esophageal adenocarcinoma, head-neck squamous cell carcinoma, ovarian cancer, stomach adenocarcinoma, and uterine corpus endometrial carcinoma. GCG expression did not significantly influence survival outcomes in bladder cancer, esophageal squamous cell carcinoma, renal papillary cell carcinoma, lung adenocarcinoma, and pancreatic cancer.

The dual role of GLP1R expression in cancer survival highlights the complexity of GLP-1 signaling in cancer biology [8]. GLP-1R is expressed at both the mRNA and protein levels across a diverse array of tissues, including the lungs, vasculature, pancreatic islets and acini, gastrointestinal tract, kidneys, gonads, urogenital system, and thyroid [40, 52]. However, the expression levels can vary significantly between these different tissues [40, 52]. Cancers arising from these tissues frequently exhibit altered GLP-1R expression [33, 35, 40]. In some cancers, such as those in the bladder, increased GLP1R expression may be protective, potentially reflecting a beneficial response to GLP-1R activation. This could be due to mechanisms such as the anti-inflammatory effects of GLP-1 agonists, their influence on metabolic pathways, or their ability to modulate cancer cell proliferation and apoptosis. On the other hand, the association between high GLP1R expression and decreased survival in cancers like cervical squamous cell carcinoma and lung squamous cell carcinoma suggests that GLP-1R activation may promote tumor progression in these contexts. This might involve pathways related to cell growth and survival that are differentially regulated in these tumor types. The observation that GLP1R expression has no effect on survival in several other cancers suggests that the impact of GLP-1R signaling is highly context-dependent and may vary based on the tumor microenvironment, genetic mutations, or other factors. The data on GCG expression further complicate this picture, showing both protective and harmful associations with cancer survival, depending on the cancer type.

In vitro and preclinical studies highlight the complexity of GLP-1 signaling in cancer biology. For instance, GLP-1R agonists like liraglutide have been shown to slow the progression of cholangiocarcinoma by inhibiting cell migration and reducing tumor growth, despite the association of GLP-1R expression with poor histological grading in intrahepatic cholangiocarcinoma tissues [10]. Additionally, GLP-1R activation inhibits glioma cell migration, invasion, and epithelial-to-mesenchymal transition, [22] as well as suppresses the growth and promotes apoptosis in ovarian [28] and prostate cancer cells [34]. Lentiviral overexpression of GLP-1R has also been demonstrated to attenuate prostate cancer cell proliferation by inhibiting cell cycle progression [20]. Moreover, GLP-1R agonists, including liraglutide, have been shown to inhibit proliferation, cell cycle progression, and/or migration in lung [13], pancreatic [31, 32], prostate [26], endometrial [15, 23], and murine colon cancer cells [38], although they were reported to promote the proliferation of breast cancer cells [14]. Contrarily, other studies have indicated that GLP-1R agonists do not affect the growth or survival of human pancreatic [53], colon [25], or thyroid cancer cells [27]. Furthermore, liraglutide has been shown to enhance the chemosensitivity of pancreatic cancer cells to gemcitabine [18] and prostate cancer cells to enzalutamide [19], and to increase radiosensitivity in prostate cancer cells through AMPK activation and subsequent inhibition of p-mTOR, cyclin B, and p34cdc2 activation [24]. Notably, both liraglutide and the GLP-1 receptor agonist exenatide significantly increased intestinal growth in healthy mice without promoting dysplasia or tumor formation in the colons of carcinogen-treated mice [37]. These findings underscore the multifactorial nature of cancer and the need to understand the specific roles that GLP-1 signaling plays in different tumorigenic processes.

Our findings are consistent with existing literature that shows a mixed impact of GLP-1 receptor agonists on cancer risk and outcomes in humans [5, 8]. For instance, in the study by Wang et al., a retrospective cohort design was employed to investigate the impact of GLP-1 receptor agonists on the incidence of 13 obesity-associated cancers in patients with type 2 diabetes [3]. The study analyzed data from over 1.6 million patients, comparing those treated with GLP-1 receptor agonists against those treated with insulin or metformin. The findings revealed that the use of GLP-1 receptor agonists was associated with a significant reduction in the risk of several cancers compared to insulin. Specifically, patients using GLP-1 receptor agonists exhibited a lower risk of esophageal, colorectal, endometrial, gallbladder, kidney, liver, ovarian, and pancreatic cancers, as well as meningioma and multiple myeloma [3]. In contrast, compared to metformin, GLP-1 receptor agonists did not demonstrate a significant reduction in cancer risk and were associated with an increased risk of kidney cancer [3]. Interestingly, in our study, high GLP-1R expression was not associated with poorer survival for either type of kidney cancer studied. These results suggest that while GLP-1 receptor agonists may offer protective effects against certain obesity-related cancers, particularly when compared to insulin, their impact varies by cancer type and may be less favorable compared to metformin. The findings also imply that insulin use could potentially promote cancer growth, underscoring the importance of considering cancer risk when selecting antidiabetic therapies. A recent study by Bukavina et al. [5] analyzed a large claims-based clinical data set to investigate the association between GLP-1 receptor agonist use and the incidence of urologic cancers, including prostate cancer, kidney cancer, and bladder cancer, in patients with T2DM. The analysis, which included data from 1,276,762 patients prescribed various antidiabetic medications, revealed that GLP-1 receptor agonist use was associated with a higher risk of bladder cancer compared to SGLT2 inhibitors (HR 1.47), kidney cancer compared to metformin (HR 1.45), and prostate cancer compared to insulin (HR 1.32) [5]. These findings suggest a potential increased risk of urologic malignancies with GLP-1 receptor agonists. Interestingly, in our study, high GLP-1R expression was associated with significantly increased survival in both bladder carcinoma and renal clear cell carcinoma, while it had no effect on survival in renal papillary cell carcinoma. Other studies have also reported that the use of sitagliptin and exenatide significantly increases the risk of pancreatitis and pancreatic cancer compared to other therapies [39]. Additionally, Mendelian randomization studies suggest that GLP-1 receptor agonists may decrease the risk of breast cancer while potentially increasing the risk of colorectal cancer [4]. Yang et al. [54] conducted a comprehensive analysis of the U.S. Food and Drug Administration Adverse Event Reporting System (FAERS) database, spanning from 2004 to 2021, to investigate the association between glucagon-like peptide-1 receptor agonists (GLP-1RAs) and tumor-related adverse events. The study identified a total of 3,593 tumor adverse event reports linked to GLP-1RA use, with a significant proportion involving pancreatic (30.5%) and thyroid (12.9%) cancers [54]. Notably, the reporting odds ratio (ROR) for pancreatic cancer was elevated at 3.19 (95% CI: 2.95–3.45), indicating a higher reporting frequency compared to other drugs [54]. Conversely, the ROR for thyroid cancer was 1.72 (95% CI: 1.50–1.97), suggesting a less pronounced association. The study also observed that the RORs for pancreatic and thyroid cancers were higher with long-acting GLP-1RAs compared to short-acting ones [54]. These findings underscore the importance of vigilant monitoring for tumor-related adverse events in patients undergoing GLP-1RA therapy, particularly concerning pancreatic and thyroid cancers. Taken together, the results emphasize the complexity of GLP-1R signaling across different cancer types and suggest that the effects of GLP-1 receptor activation may vary widely depending on the specific tumor biology. Further investigation is necessary to validate these associations and to determine whether they are causal or influenced by confounding factors.

In contrast to concerns raised in the broader literature [6–8], our study found that higher GLP1R expression was associated with substantially better survival in patients with thyroid carcinoma. Consistent with our findings, a previous study reported that GLP-1R expression in papillary thyroid carcinomas is negatively correlated with tumor multifocality [35]. These findings are particularly intriguing given the FDA’s warnings about the potential increased risk of thyroid cancer, specifically medullary thyroid carcinoma, in patients treated with the GLP-1 receptor agonists [6, 7]. The positive correlation between high GLP1R expression and improved survival in our study suggests that, while GLP-1 receptor agonists may increase the risk of developing thyroid cancer, those tumors with higher GLP1R expression could be more responsive to GLP-1R signaling, leading to better clinical outcomes. This could imply a potential protective role of GLP1R in the context of established thyroid cancer, possibly by modulating tumor behavior [12] or enhancing the efficacy of treatment. However, the exact mechanisms underlying this relationship remain unclear, and further research is needed to fully understand how GLP1R expression influences thyroid cancer progression and survival.

This duality in GLP-1R’s role may reflect the complex interplay between the drug’s metabolic effects, its direct action on cancer cells, and the broader hormonal and environmental context within the body. Several potential mechanisms could explain the observed effects of GLP-1 agonists on cancer development and survival. These include the role of GLP-1R in regulating fibroblast growth factor 21 (FGF21) synthesis, which has been implicated in cancer biology, as well as its anti-inflammatory effects, which could suppress tumor-promoting inflammation. Weight loss induced by GLP-1 agonists may also play a protective role by reducing obesity-related cancer risks. Conversely, the promotion of insulin secretion by GLP-1 agonists could, in certain contexts, stimulate tumor growth, particularly in cancers sensitive to insulin and insulin-like growth factors.

In addition to their role in tumor biology, GLP-1R agonists have shown potential cardioprotective effects, particularly in mitigating chemotherapy-induced cardiotoxicity. This is a critical consideration for cancer patients undergoing treatment with cardiotoxic agents, such as anthracyclines. Recent studies [55, 56] highlight that GLP-1R agonists may protect against cardiotoxicity by modulating oxidative stress, inflammation, and mitochondrial dysfunction, thereby preserving cardiac function during chemotherapy.

Given the varying effects of GLP1R expression on survival across different cancer types, the use of GLP-1 receptor agonists in obese or diabetic patients at high risk for cancer should be approached with caution. Clinicians should consider the specific cancer risks associated with GLP-1R agonists and balance these against the benefits of glycemic control and weight loss. In patients with a high risk of cancers where GLP1R expression is associated with poor outcomes, alternative therapies might be more appropriate. Conversely, for patients at risk of cancers where GLP1R expression is protective, GLP-1 receptor agonists could be considered a beneficial option.

While our study provides important insights, it is crucial to recognize its limitations. Methodological factors such as variations in tumor stages, interactions with established prognostic biomarkers, differences in treatment protocols, socioeconomic factors, and the analytical methods used may introduce biases that could influence the interpretation of our findings. Additionally, the retrospective nature of our study may limit the generalizability of the results to broader patient populations. The heterogeneity of the cancers analyzed and the differing effects of GLP1R and GCG expression indicate that the relationship between GLP-1 signaling and cancer is likely more intricate than this study alone can elucidate. Future research should focus on integrating GLP1R signaling-related gene expression signatures with existing biomarkers to improve prognostic accuracy across multiple cancer types. Analyzing gene expression profiles from tumors in patients treated with GLP-1 receptor agonists and other related therapies will be vital. This approach could provide deeper insights into how these drugs interact with the tumor microenvironment and influence cancer progression, ultimately leading to more personalized and effective treatment strategies. Further, the impact of GLP-1R agonists on cancer outcomes may vary significantly based on patient age and sex, emphasizing the need to consider age- and sex-specific factors in evaluating their therapeutic use. Elderly patients may experience differential responses to GLP-1R agonists due to age-related changes in GLP1R expression, tumor microenvironment, and systemic physiology. For example, reduced regenerative capacity and altered immune function in older adults could influence both the efficacy and safety of GLP-1R agonists in managing cancer outcomes. Additionally, elderly patients often present with multiple comorbidities and polypharmacy, which could further modulate the effects of these agents. Sex-specific factors are equally important, particularly in hormone-sensitive cancers such as breast and endometrial cancers, which may exhibit unique responses to GLP-1R signaling. Emerging studies have highlighted sex-dependent variations in cancer biology and treatment responses, suggesting that GLP-1R agonists could interact differently with tumor progression pathways in male versus female patients. For instance, the influence of GLP1R expression on estrogen or progesterone receptor-positive tumors might explain differential survival outcomes in cancers like breast and uterine carcinomas. Given these considerations, future research should prioritize stratified analyses to delineate the age- and sex-specific impacts of GLP-1R agonists on cancer progression and survival. Such studies would provide valuable insights into optimizing the therapeutic use of GLP-1R agonists in diverse patient populations, ensuring personalized and effective cancer management.

In conclusion, this study provides a nuanced view of the role of GLP1R expression in cancer survival, highlighting both protective and adverse associations depending on the cancer type. These findings underscore the complex role of GLP-1 receptor agonists in cancer risk and survival and suggest that the therapeutic use of these agents should be carefully tailored to the individual patient’s cancer risk profile. Future research should focus on elucidating the molecular mechanisms underlying the differential effects of GLP1R expression in various cancers. Prospective studies are needed to confirm these findings and to explore how these effects might be modulated by other factors, such as concurrent therapies, genetic mutations, and the tumor microenvironment. Given the diverse effects of GLP1R and GCG expression on cancer survival, clinicians should exercise caution when prescribing GLP-1 receptor agonists, particularly in patients with known cancer risks. Tailored treatment plans that consider both the metabolic benefits and potential oncological risks of GLP-1 receptor agonists are essential for optimizing patient outcomes.

## References

[1] Campbell JE, Muller TD, Finan B, DiMarchi RD, Tschop MH, D’Alessio DA. GIPR/GLP-1R dual agonist therapies for diabetes and weight loss-chemistry, physiology, and clinical applications. Cell Metab. 2023;35:1519–29. 10.1016/j.cmet.2023.07.010. 37591245 10.1016/j.cmet.2023.07.010 PMC10528201

[2] Tahrani AA, Barnett AH, Bailey CJ. Pharmacology and therapeutic implications of current drugs for type 2 diabetes mellitus. Nat Rev Endocrinol. 2016;12:566–92. 10.1038/nrendo.2016.86. 27339889 10.1038/nrendo.2016.86

[3] Wang L, Xu R, Kaelber DC, Berger NA. Glucagon-like peptide 1 receptor agonists and 13 obesity-associated cancers in patients with type 2 diabetes. JAMA Netw Open. 2024;7: e2421305. 10.1001/jamanetworkopen.2024.21305. 38967919 10.1001/jamanetworkopen.2024.21305 PMC11227080

[4] Sun Y, Liu Y, Dian Y, Zeng F, Deng G, Lei S. Association of glucagon-like peptide-1 receptor agonists with risk of cancers-evidence from a drug target Mendelian randomization and clinical trials. Int J Surg. 2024;110:4688–94. 10.1097/JS9.0000000000001514. 38701500 10.1097/JS9.0000000000001514 PMC11325911

[5] Bukavina L, Helstrom E, Wallis CJD, Fulmes A, Calaway A, Correa A, Rhodes S. Association between GLP1R agonists and prostate, kidney, and bladder cancers. Eur Urol Oncol. 2024. 10.1016/j.euo.2024.04.006. 38702255 10.1016/j.euo.2024.04.006

[6] FDA. Rybelsus - Full Prescribing Information. https://www.accessdata.fda.gov/drugsatfda_docs/label/2023/213051s012lbl.pdf accessed on 08/22/2024.

[7] FDA. Osempic - Full Prescribing Information. https://www.accessdata.fda.gov/drugsatfda_docs/label/2022/209637s012lbl.pdf accessed on 08/22/2024.

[8] Vangoitsenhoven R, Mathieu C, Van der Schueren B. GLP1 and cancer: friend or foe? Endocr Relat Cancer. 2012;19:F77-88. 10.1530/ERC-12-0111. 22691625 10.1530/ERC-12-0111

[9] Ibrahim SS, Ibrahim RS, Arabi B, Brockmueller A, Shakibaei M, Busselberg D. The effect of GLP-1R agonists on the medical triad of obesity, diabetes, and cancer. Cancer Metastasis Rev. 2024. 10.1007/s10555-024-10192-9. 38801466 10.1007/s10555-024-10192-9 PMC11554930

[10] Trakoonsenathong R, Kunprom W, Aphivatanasiri C, Yueangchantuek P, Pimkeeree P, Sorin S, Khawkhiaw K, Chiu CF, Okada S, Wongkham S, Saengboonmee C. Liraglutide exhibits potential anti-tumor effects on the progression of intrahepatic cholangiocarcinoma, in vitro and in vivo. Sci Rep. 2024;14:13726. 10.1038/s41598-024-64774-2. 38877189 10.1038/s41598-024-64774-2 PMC11178799

[11] Stein MS, Kalff V, Williams SG, Murphy DG, Colman PG, Hofman MS. The GLP-1 receptor is expressed in vivo by human metastatic prostate cancer. Endocr Oncol. 2024;4: e230015. 10.1530/EO-23-0015. 38313829 10.1530/EO-23-0015 PMC10831528

[12] Zhang X, Zhang L, Wang B, Zhang X, Gu L, Guo K, Zhang X, Zhou Z. GLP-1 receptor agonist liraglutide inhibits the proliferation and migration of thyroid cancer cells. Cell Mol Biol (Noisy-le-grand). 2023;69:221–5. 10.14715/cmb/2023.69.14.37. 38279433 10.14715/cmb/2023.69.14.37

[13] Pu Z, Yang Y, Qin S, Li X, Cui C, Chen W. The effect of liraglutide on lung cancer and its potential protective effect on high glucose-induced lung senescence and oxidative damage. Front Biosci (Landmark Ed). 2023;28:259. 10.31083/j.fbl2810259. 37919054 10.31083/j.fbl2810259

[14] Liu ZZ, Duan XX, Yuan MC, Yu J, Hu X, Han X, Lan L, Liu BW, Wang Y, Qin JF. Glucagon-like peptide-1 receptor activation by liraglutide promotes breast cancer through NOX4/ROS/VEGF pathway. Life Sci. 2022;294: 120370. 10.1016/j.lfs.2022.120370. 35124000 10.1016/j.lfs.2022.120370

[15] Li W, Gu Y, Liu S, Ruan F, Lv W. GLP1R inhibits the progression of endometrial carcinoma through activation of cAMP/PKA pathway. J Clin Lab Anal. 2022;36: e24604. 10.1002/jcla.24604. 35989517 10.1002/jcla.24604 PMC9551121

[16] Abdul-Maksoud RS, Elsayed WSH, Rashad NM, Elsayed RS, Elshorbagy S, Hamed MG. GLP-1R polymorphism (rs1042044) and expression are associated with the risk of papillary thyroid cancer among the Egyptian population. Gene. 2022;834: 146597. 10.1016/j.gene.2022.146597. 35598685 10.1016/j.gene.2022.146597

[17] Mao D, Cao H, Shi M, Wang CC, Kwong J, Li JJX, Hou Y, Ming X, Lee HM, Tian XY, Wong CK, Chow E, Kong APS, Lui VWY, Chan PKS, Chan JCN. Increased co-expression of PSMA2 and GLP-1 receptor in cervical cancer models in type 2 diabetes attenuated by Exendin-4: A translational case-control study. EBioMedicine. 2021;65: 103242. 10.1016/j.ebiom.2021.103242. 33684886 10.1016/j.ebiom.2021.103242 PMC7938253

[18] Zhao HJ, Jiang X, Hu LJ, Yang L, Deng LD, Wang YP, Ren ZP. Activation of GLP-1 receptor enhances the chemosensitivity of pancreatic cancer cells. J Mol Endocrinol. 2020;64:103–13. 10.1530/JME-19-0186. 31855560 10.1530/JME-19-0186

[19] Wenjing H, Shao Y, Yu Y, Huang W, Feng G, Li J. Exendin-4 enhances the sensitivity of prostate cancer to enzalutamide by targeting Akt activation. Prostate. 2020;80:367–75. 10.1002/pros.23951. 31967357 10.1002/pros.23951

[20] Shigeoka T, Nomiyama T, Kawanami T, Hamaguchi Y, Horikawa T, Tanaka T, Irie S, Motonaga R, Hamanoue N, Tanabe M, Nabeshima K, Tanaka M, Yanase T, Kawanami D. Activation of overexpressed glucagon-like peptide-1 receptor attenuates prostate cancer growth by inhibiting cell cycle progression. J Diabetes Investig. 2020;11:1137–49. 10.1111/jdi.13247. 32146725 10.1111/jdi.13247 PMC7477521

[21] Li M, Liu Y, Xu Y, Li Y, Pan D, Wang L, Yan J, Wang X, Yang R, Yang M. Preliminary evaluation of GLP-1R PET in the diagnosis and risk stratification of pheochromocytomas. Neoplasma. 2020;67:27–36. 10.4149/neo_2019_190227N163. 31686522 10.4149/neo_2019_190227N163

[22] Nie ZJ, Zhang YG, Chang YH, Li QY, Zhang YL. Exendin-4 inhibits glioma cell migration, invasion and epithelial-to-mesenchymal transition through GLP-1R/sirt3 pathway. Biomed Pharmacother. 2018;106:1364–9. 10.1016/j.biopha.2018.07.092. 30119208 10.1016/j.biopha.2018.07.092

[23] Kanda R, Hiraike H, Wada-Hiraike O, Ichinose T, Nagasaka K, Sasajima Y, Ryo E, Fujii T, Osuga Y, Ayabe T. Expression of the glucagon-like peptide-1 receptor and its role in regulating autophagy in endometrial cancer. BMC Cancer. 2018;18:657. 10.1186/s12885-018-4570-8. 29907137 10.1186/s12885-018-4570-8 PMC6003019

[24] He W, Li J. Exendin-4 enhances radiation response of prostate cancer. Prostate. 2018;78:1125–33. 10.1002/pros.23687. 30009503 10.1002/pros.23687

[25] Wenjing H, Shuang Y, Weisong L, Haipeng X. Exendin-4 does not modify growth or apoptosis of human colon cancer cells. Endocr Res. 2017;42:209–18. 10.1080/07435800.2017.1292525. 28318339 10.1080/07435800.2017.1292525

[26] Li XN, Bu HM, Ma XH, Lu S, Zhao S, Cui YL, Sun J. Glucagon-like peptide-1 analogues inhibit proliferation and increase apoptosis of human prostate cancer cells in vitro. Exp Clin Endocrinol Diabetes. 2017;125:91–7. 10.1055/s-0042-112368. 28008585 10.1055/s-0042-112368

[27] He L, Zhang S, Zhang X, Liu R, Guan H, Zhang H. Effects of insulin analogs and glucagon-like peptide-1 receptor agonists on proliferation and cellular energy metabolism in papillary thyroid cancer. Onco Targets Ther. 2017;10:5621–31. 10.2147/OTT.S150701. 29200876 10.2147/OTT.S150701 PMC5703165

[28] He W, Yu S, Wang L, He M, Cao X, Li Y, Xiao H. Exendin-4 inhibits growth and augments apoptosis of ovarian cancer cells. Mol Cell Endocrinol. 2016;436:240–9. 10.1016/j.mce.2016.07.032. 27496641 10.1016/j.mce.2016.07.032

[29] Dal Molin M, Kim H, Blackford A, Sharma R, Goggins M. Glucagon-like peptide-1 receptor expression in normal and neoplastic human pancreatic tissues. Pancreas. 2016;45:613–9. 10.1097/MPA.0000000000000521. 26495786 10.1097/MPA.0000000000000521 PMC4783303

[30] Waser B, Blank A, Karamitopoulou E, Perren A, Reubi JC. Glucagon-like-peptide-1 receptor expression in normal and diseased human thyroid and pancreas. Mod Pathol. 2015;28:391–402. 10.1038/modpathol.2014.113. 25216224 10.1038/modpathol.2014.113

[31] Zhao H, Wei R, Wang L, Tian Q, Tao M, Ke J, Liu Y, Hou W, Zhang L, Yang J, Hong T. Activation of glucagon-like peptide-1 receptor inhibits growth and promotes apoptosis of human pancreatic cancer cells in a cAMP-dependent manner. Am J Physiol Endocrinol Metab. 2014;306:E1431-1441. 10.1152/ajpendo.00017.2014. 24801389 10.1152/ajpendo.00017.2014

[32] Zhao H, Wang L, Wei R, Xiu D, Tao M, Ke J, Liu Y, Yang J, Hong T. Activation of glucagon-like peptide-1 receptor inhibits tumourigenicity and metastasis of human pancreatic cancer cells via PI3K/Akt pathway. Diabetes Obes Metab. 2014;16:850–60. 10.1111/dom.12291. 24641303 10.1111/dom.12291

[33] Wada R, Yagihashi S. The expression of glucagon-like peptide-1 receptor and dipeptidyl peptidase-IV in neuroendocrine neoplasms of the pancreas and gastrointestinal tract. Endocr Pathol. 2014;25:390–6. 10.1007/s12022-014-9326-7. 25119061 10.1007/s12022-014-9326-7

[34] Nomiyama T, Kawanami T, Irie S, Hamaguchi Y, Terawaki Y, Murase K, Tsutsumi Y, Nagaishi R, Tanabe M, Morinaga H, Tanaka T, Mizoguchi M, Nabeshima K, Tanaka M, Yanase T. Exendin-4, a GLP-1 receptor agonist, attenuates prostate cancer growth. Diabetes. 2014;63:3891–905. 10.2337/db13-1169. 24879833 10.2337/db13-1169

[35] Jung MJ, Kwon SK. Expression of glucagon-like Peptide-1 receptor in papillary thyroid carcinoma and its clinicopathologic significance. Endocrinol Metab (Seoul). 2014;29:536–44. 10.3803/EnM.2014.29.4.536. 25559577 10.3803/EnM.2014.29.4.536 PMC4285044

[36] Reubi JC. Old and new peptide receptor targets in cancer: future directions. Recent Results Cancer Res. 2013;194:567–76. 10.1007/978-3-642-27994-2_34. 22918784 10.1007/978-3-642-27994-2_34

[37] Kissow H, Hartmann B, Holst JJ, Viby NE, Hansen LS, Rosenkilde MM, Hare KJ, Poulsen SS. Glucagon-like peptide-1 (GLP-1) receptor agonism or DPP-4 inhibition does not accelerate neoplasia in carcinogen treated mice. Regul Pept. 2012;179:91–100. 10.1016/j.regpep.2012.08.016. 22989472 10.1016/j.regpep.2012.08.016

[38] Koehler JA, Kain T, Drucker DJ. Glucagon-like peptide-1 receptor activation inhibits growth and augments apoptosis in murine CT26 colon cancer cells. Endocrinology. 2011;152:3362–72. 10.1210/en.2011-1201. 21771884 10.1210/en.2011-1201

[39] Elashoff M, Matveyenko AV, Gier B, Elashoff R, Butler PC. Pancreatitis, pancreatic, and thyroid cancer with glucagon-like peptide-1-based therapies. Gastroenterology. 2011;141:150–6. 10.1053/j.gastro.2011.02.018. 21334333 10.1053/j.gastro.2011.02.018 PMC4404515

[40] Korner M, Stockli M, Waser B, Reubi JC. GLP-1 receptor expression in human tumors and human normal tissues: potential for in vivo targeting. J Nucl Med. 2007;48:736–43. 10.2967/jnumed.106.038679. 17475961 10.2967/jnumed.106.038679

[41] Gyorffy B. Transcriptome-level discovery of survival-associated biomarkers and therapy targets in non-small-cell lung cancer. Br J Pharmacol. 2024;181:362–74. 10.1111/bph.16257. 37783508 10.1111/bph.16257

[42] Gyorffy B, Surowiak P, Budczies J, Lanczky A. Online survival analysis software to assess the prognostic value of biomarkers using transcriptomic data in non-small-cell lung cancer. PLoS ONE. 2013;8: e82241. 10.1371/journal.pone.0082241. 24367507 10.1371/journal.pone.0082241 PMC3867325

[43] Ungvari Z, Ungvari A, Bianchini G, Gyorffy B. Prognostic significance of a signature based on senescence-related genes in colorectal cancer. Geroscience. 2024. 10.1007/s11357-024-01164-6. 38658505 10.1007/s11357-024-01164-6 PMC11336146

[44] Bartha A, Gyorffy B. TNMplot.com: a web tool for the comparison of gene expression in normal, tumor and metastatic tissues. Int J Mol Sci. 2021;22:2622. 10.3390/ijms22052622. 33807717 10.3390/ijms22052622 PMC7961455

[45] Gyorffy B. Survival analysis across the entire transcriptome identifies biomarkers with the highest prognostic power in breast cancer. Comput Struct Biotechnol J. 2021;19:4101–9. 10.1016/j.csbj.2021.07.014. 34527184 10.1016/j.csbj.2021.07.014 PMC8339292

[46] Gyorffy B. Integrated analysis of public datasets for the discovery and validation of survival-associated genes in solid tumors. Innovation (Camb). 2024;5: 100625. 10.1016/j.xinn.2024.100625. 38706955 10.1016/j.xinn.2024.100625 PMC11066458

[47] Gyorffy B. Discovery and ranking of the most robust prognostic biomarkers in serous ovarian cancer. Geroscience. 2023;45:1889–98. 10.1007/s11357-023-00742-4. 36856946 10.1007/s11357-023-00742-4 PMC10400493

[48] Szasz AM, Lanczky A, Nagy A, Forster S, Hark K, Green JE, Boussioutas A, Busuttil R, Szabo A, Gyorffy B. Cross-validation of survival associated biomarkers in gastric cancer using transcriptomic data of 1,065 patients. Oncotarget. 2016;7:49322–33. 10.18632/oncotarget.10337. 27384994 10.18632/oncotarget.10337 PMC5226511

[49] Posta M, Gyorffy B. Analysis of a large cohort of pancreatic cancer transcriptomic profiles to reveal the strongest prognostic factors. Clin Transl Sci. 2023;16:1479–91. 10.1111/cts.13563. 37260110 10.1111/cts.13563 PMC10432876

[50] Lanczky A, Gyorffy B. Web-based survival analysis tool tailored for medical research (KMplot): development and implementation. J Med Internet Res. 2021;23: e27633. 10.2196/27633. 34309564 10.2196/27633 PMC8367126

[51] Li Q, Birkbak NJ, Gyorffy B, Szallasi Z, Eklund AC. Jetset: selecting the optimal microarray probe set to represent a gene. BMC Bioinformatics. 2011;12:474. 10.1186/1471-2105-12-474. 22172014 10.1186/1471-2105-12-474 PMC3266307

[52] Waser B, Reubi JC. Radiolabelled GLP-1 receptor antagonist binds to GLP-1 receptor-expressing human tissues. Eur J Nucl Med Mol Imaging. 2014;41:1166–71. 10.1007/s00259-013-2684-4. 24519555 10.1007/s00259-013-2684-4

[53] Koehler JA, Drucker DJ. Activation of glucagon-like peptide-1 receptor signaling does not modify the growth or apoptosis of human pancreatic cancer cells. Diabetes. 2006;55:1369–79. 10.2337/db05-1145. 16644694 10.2337/db05-1145

[54] Yang Z, Lv Y, Yu M, Mei M, Xiang L, Zhao S, Li R. GLP-1 receptor agonist-associated tumor adverse events: a real-world study from 2004 to 2021 based on FAERS. Front Pharmacol. 2022;13: 925377. 10.3389/fphar.2022.925377. 36386208 10.3389/fphar.2022.925377 PMC9640975

[55] Atef MM, Hafez YM, El-Deeb OS, Basha EH, Ismail R, Alshenawy H, El-Esawy RO, Eltokhy AK. The cardioprotective effect of human glucagon-like peptide-1 receptor agonist (semaglutide) on cisplatin-induced cardiotoxicity in rats: targeting mitochondrial functions, dynamics, biogenesis, and redox status pathways. Cell Biochem Funct. 2023;41:450–60. 10.1002/cbf.3795. 37051656 10.1002/cbf.3795

[56] Li X, Luo W, Tang Y, Wu J, Zhang J, Chen S, Zhou L, Tao Y, Tang Y, Wang F, Huang Y, Jose PA, Guo L, Zeng C. Semaglutide attenuates doxorubicin-induced cardiotoxicity by ameliorating BNIP3-Mediated mitochondrial dysfunction. Redox Biol. 2024;72: 103129. 10.1016/j.redox.2024.103129. 38574433 10.1016/j.redox.2024.103129 PMC11000183

